# SNPchiMp v.3: integrating and standardizing single nucleotide polymorphism data for livestock species

**DOI:** 10.1186/s12864-015-1497-1

**Published:** 2015-04-10

**Authors:** Ezequiel L Nicolazzi, Andrea Caprera, Nelson Nazzicari, Paolo Cozzi, Francesco Strozzi, Cindy Lawley, Ali Pirani, Chandrasen Soans, Fiona Brew, Hossein Jorjani, Gary Evans, Barry Simpson, Gwenola Tosser-Klopp, Rudiger Brauning, John L Williams, Alessandra Stella

**Affiliations:** Bioinformatics and Biostatistical Genomics group, Fondazione Parco Tecnologico Padano, Via Einstein, Loc. Cascina Codazza, 26900 Lodi, Italy; Illumina Inc, 5200 Illumina Way, San Diego, CA 92121 USA; Affymetrix Inc, 3420 Central Expressway, Santa Clara, CA 95051 USA; Affymetrix UK Ltd, Mercury Park, Wycombe Lane, High Wycombe, HP10 0HH UK; Interbull center, Uppsala, S-75007 Sweden; GeneSeek, a Neogen Company, Auchincruive, Ayr KA6 5HU Scotland, UK; GeneSeek, a Neogen Company, Lincoln, NE 68504 USA; Génétique, Physiologie et Systèmes d’Élevage, Institut National de la Recherche Agronomique & Ecole Nationale Vétérinaire de Toulouse & Ecole Nationale Supérieure Agronomique de Toulouse, Castanet-Tolosan, 31326 France; AgResearch, Invermay Agricultural Centre, PB 50034 Mosgiel, New Zealand; Istituto di Biologia e Biotecnologia Agraria, Consiglio Nazionale delle Ricerche, Via Einstein, Cascina Codazza, Lodi 26900 Italy

**Keywords:** SNP, Array, Illumina, Affymetrix, GeneSeek, Integration, Standardization, Imputation, Ensembl, NCBI

## Abstract

**Background:**

In recent years, the use of genomic information in livestock species for genetic improvement, association studies and many other fields has become routine. In order to accommodate different market requirements in terms of genotyping cost, manufacturers of single nucleotide polymorphism (SNP) arrays, private companies and international *consortia* have developed a large number of arrays with different content and different SNP density. The number of currently available SNP arrays differs among species: ranging from one for goats to more than ten for cattle, and the number of arrays available is increasing rapidly. However, there is limited or no effort to standardize and integrate array- specific (e.g. SNP IDs, allele coding) and species-specific (i.e. past and current assemblies) SNP information.

**Results:**

Here we present SNPchiMp v.3, a solution to these issues for the six major livestock species (cow, pig, horse, sheep, goat and chicken). Original data was collected directly from SNP array producers and specific international genome consortia, and stored in a MySQL database. The database was then linked to an open-access web tool and to public databases. SNPchiMp v.3 ensures fast access to the database (retrieving within/across SNP array data) and the possibility of annotating SNP array data in a user-friendly fashion.

**Conclusions:**

This platform allows easy integration and standardization, and it is aimed at both industry and research. It also enables users to easily link the information available from the array producer with data in public databases, without the need of additional bioinformatics tools or pipelines. In recognition of the open-access use of Ensembl resources, SNPchiMp v.3 was officially credited as an Ensembl E!mpowered tool. Availability at http://bioinformatics.tecnoparco.org/SNPchimp.

## Background

In Europe, agriculture represents approximately 1.7% of the total added gross value [1]. This share differs greatly among member countries, ranging from 0.9% in Luxembourg to 13% in Romania. European livestock output is estimated to be worth approximately 165 billion Euros [2] per year. Although the number of animals for all livestock species has decreased steadily since the 1980, increased farming efficiency and genetic improvement of animals have led to overall increasing yields. Research on genetic characteristics of livestock species has increased since the availability of genome sequences of major livestock species and the consequent availability of SNP arrays. Livestock genetic improvement, breeding, conservation and many other related sectors now rely on SNP array technology, as it provides rapid access to highly accurate genome-wide information on individuals at relatively low cost. Thousands of individuals have been genotyped worldwide [3-6], and this information has been used mainly for selection in breeding programmes. However, the amount of genomic information available on each animal differs depending on the type of genotyping array used. Furthermore, the number of SNP arrays available is rapidly increasing. It is common practice within all major livestock species to periodically exchange genotype data, which might be obtained from different SNP arrays. Imputation methods may then be used to predict the missing data [7,8]. Although exchanging information is routine, the difficulties in comparing and merging data is usually an underappreciated burden. There is an urgent need for standardization and tools to integrate SNP data and related information. In addition accessory information including annotation, allele formats, flanking sequences, etc. is often difficult to retrieve, especially for SNP arrays that are no longer commercially available. Integration and standardization difficulties are compounded as basic information such as SNP IDs (e.g. names) are not consistent across array suppliers, and in some cases, not even between array releases from the same supplier. In addition, SNP IDs are not always searchable in public databases and in particular Ensembl and NCBI. Even when SNP related information is available, it often requires the download of large datasets and extensive data manipulation to obtain the required information. Moreover, reference genome assemblies are regularly updated, with consequent changes in SNP coordinates and occasionally in the allele orientation coding. Cattle is the most extreme example for this, where there are currently two reference genome assemblies (UMD3.1 and BTAU4.6) [9,10] being used in parallel. SNP producers usually provide SNP coordinates quoting the UMD3.1 assembly. The cattle genome was one of the first livestock genomes to be published [11] and the subsequent releases have greatly increased quality and stability of the sequence. The reference genome assemblies on other livestock species are more recent and still relatively unstable [12], with ongoing work to improve the draft assemblies. The SNPchiMP v.3 presented here is the first tool able to tackle all the above problems for livestock species in an integrated, user-friendly and open-access fashion.

## Implementation

### Data

The SNP data contained in SNPchiMp v.3 can be divided into two main categories: the original data coming from the producer and the information from public databases. Current and historical original data free from intellectual property was collected directly from producers (Illumina, Neogen-GeneSeek and Affymetrix) and International genome consortia (the International Sheep Genomics Consortium [13] and the International Goat Genome Consortium [14]). Some of the information contained in SNPchiMp v.3 is directly available from the manufacturers/consortia websites [15,16].

As a result of extensive data collection, a total of 23 SNP arrays (12 cow, 4 pig, 3 horse, 2 sheep, 1 goat and 1 chicken) are currently stored in SNPchiMP, with a total of 3,993,814 SNP and their cross-references.

Public database information was handled in two ways: i) dbSNP database builds from June 2012 [17,18] onwards for all six livestock species were downloaded and stored on a local server; ii) tools to interrogate Ensembl databases directly through the web were built, either for querying information for single SNP or to access the Ensembl Biomart [19,20] and/or VeP [21,22] tools.

A key feature of SNPchiMp, which is essential to integrate all above sources of information, was obtaining the links between commercial SNP IDs and dbSNP RefSeq (rs) IDs. Affymetrix already provides such association in its SNP knowledge base, whereas all other companies and *consortia*, which use Illumina-based technology and software, do not provide such information. For these, associations for all species were obtained by matching commercial SNP IDs against the “Submitter ID” or flanking sequences in the locally stored dbSNP database, following a similar procedure as specified in [23] for cattle. However, full flanking sequence BLAST [24] against the reference genomes of the six species was not performed, as this procedure was proven to be considerably time- and resource-consuming, and redundant with respect to independent checks performed over the whole dataset.

### Database

**Figure 1:**
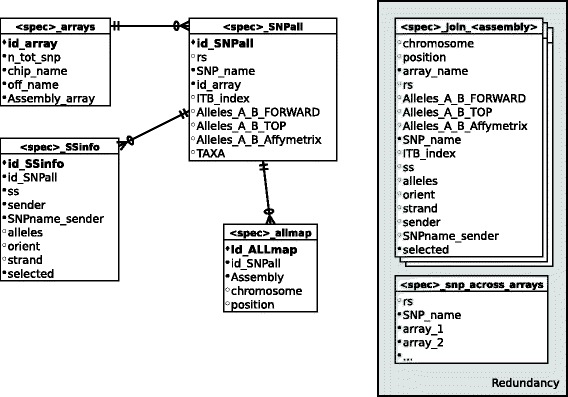
Basic entity-relationship model for each species in the tool. Primary keys of each table are in bold. Black circles indicate required fields (e.g. non-NULL).

SNPchiMp v.3 stores the data on a MySQL Server instance version 5.1.73. Data are internally organized as shown in the entity-relationship shown in Figure 1. The different species were considered separately, by replicating the same set of tables for each using a naming scheme (e.g. prefix " < spec > " for each species). The core data was simplified in order to require only four tables:

- <spec > _arrays contains general SNP array information, including official SNP array name, the assembly used for the design, and the number of SNP contained.
- <spec > _SNPall contains the original SNP information provided by producers for all arrays. This table includes commercial SNP names and rs IDs (if assigned), all possible alleles coding for that SNP, the Interbull index (if available; only for cattle) and a code for the taxa (for internal control).
- <spec > _ALLmap contains all available assembly mapping for all SNPs. As such, it is in a one-to-many relationship with < spec > _SNPall, as a SNP can be mapped on more than one assembly (and more than one position on a different assembly).
- <spec > _SSinfo contains SNP IDs submitted to dbSNP (ss ID). This table is in a one-to-many relationship with < org > _SNPall, as each rs ID usually contains several ss IDs (e.g. the same SNP was submitted by multiple groups). A Boolean flag (i.e. true/false) was added to identify which of the ss IDs was used to link the commercial SNP name to the RefSeq ID.

Two additional redundant tables were created for each species, for performance purposes:

- <spec > _snp_across_arrays records the presence (or absence) of SNP across arrays. As such, the table structure is species-specific and the number of columns differs depending on the number of available SNP arrays. The table has two columns that describe the SNP (commercial SNP names and, if available, rs ID) and one Boolean column for each array, recording the presence/absence of that SNP in the specified array, respectively.
- <spec > _join_ < assembly > is a set of tables. For each species, there are as many of these tables as the number of available assemblies. Each table represents a join between the four core tables, restricted to a single assembly. This data design enhances data compartmentalization with respect to assemblies.

### Query engine

**Figure 2:**
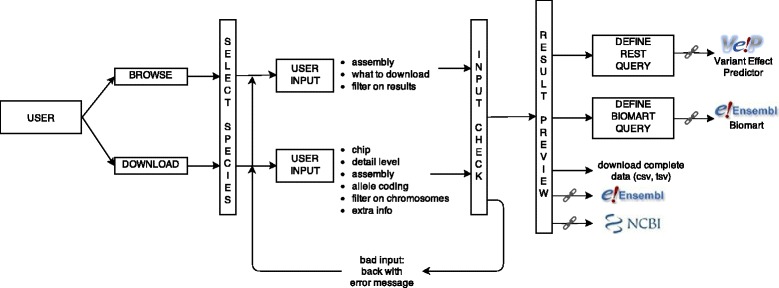
Schematic view of query engine and user interface functionalities.

The query engine relies on a PHP application programming interface (API) written *ad hoc* to facilitate dynamically built SQL queries of the SNPchiMp v.3 database (Figure 2). Each user input is validated and translated into a SQL query. A conservative approach was applied to ensure the security of the information contained in the database, such that a user request that fails validation returns an error status and a set of case-specific error messages. For valid requests, the engine works in two different modes: one to produce a “preview” of the data requested by the user and one to perform the full query of the database. In the first mode, the engine interrogates the MySQL database, parses a subset of 25 elements of the result and returns a properly formatted PHP object. In the second mode, when a full query is requested, the engine returns the mediated user-defined SQL query using a pipeline, which directly connects the database output to the user. This facilitates massive downloads of data to the web-server with no need for pre-processing.

Extensive testing is performed before releasing new information. This is carried out using *ad hoc* development environments built specifically to avoid any conflict or unpredicted modifications to the production environment, which is publicly accessible.

## Results and discussion

The tool presented here is the evolution of two previous versions. SNPchiMp v.1 [23] was specifically focused on bovine cattle and contained only 6 SNP arrays. SNPchiMp v.2 [25] was a first attempt to enlarge the included set of species and SNP arrays. These two previous versions of SNPchimp were progressive upgrades, useful to gradually improve the tool and to generate interest among stakeholders and researchers. Since then, the strong interaction and collaboration with major industries and international genome consortia was the basis of the version presented here. In fact, SNPchiMp v.3 includes a notable growth in the amount of data regarding new species and chips (e.g. nearly two-fold increase from version 2). More than just a quantitative improvement, this was also a major qualitative change allowing a wider audience addressing common problems in multiple species. From the technological point of view, the whole tool has been completely rebuilt. Performance, database structure and technology and languages behind the user interface were highly improved to allow current and future scalability and reliability. In addition, this version includes a completely new functionality for direct interrogation of the BioMart and VeP Ensembl resources (i.e. for SNP array automatic annotation).

The key to ensure data reliability was to obtain current and historical original data directly from producers and international genome consortia. As SNPchiMp v.3 aimed at the animal genetics community as a whole, only SNP arrays (or SNP information) that were free from intellectual property (IP) were considered. Once the data was obtained, a specific design of the MySQL database was chosen. The strong compartmentalization between different species was one of the most important design choices. This ensured smaller table size, and thus easier updates and additions. Moreover, it reduced the probability that corruption in one dataset propagates to others, thus providing higher structure stability. In any case, the original data was stored in the database without modifying the data itself (i.e. data was just integrated, standardized and arranged to a MySQL database format), thus users are not forced to store large annotation files anymore. In fact, users do not need to build cross-reference or cross-SNP array files anymore, as all these are already included in the functionalities of this tool (using original information). Another important design choice was the creation of two (controlled) redundant tables per species. These tables were useful to enhance the performance of the most common queries (by reducing the number of “joins” that need to be performed) and, hence, runtime.

The choice of producing a “preview” page of the queries was also linked to high performance, as PHP objects are easy to render graphically and parsing time is very short. Specifically, the “preview” functionality was included to reduce the number of unwanted downloads, which would be both tedious for the user and resource-consuming for the server.

A strong effort was put into the design of an appealing, user-friendly and easy-to-manage website that included the interface to interrogate the database. Differently from previous versions, the SNPchiMp v.3 website was built using the Joomla content management system (CMS) v.3.2.2 [26]. It was functionally and structurally divided in three main areas: a documentation section, a software section and the database interface.

The documentation area was highly improved from previous versions. Now it contains general information on the SNPchiMp, the data used, news with an RSS feed (Resource description framework Site Summary) and contact details.

The new software section (“Tools”) consists of a webpage that points to useful tools, usually linked to SNPchiMp v.3 outputs (but not necessarily) and either produced in-house or by third parties. All software present in this section is open-access, and all contributors are encouraged to make their scripts open-source. Currently, the Tools menu contains software to extract Affymetrix Axiom genotypes (AffyPipe) [27], to convert Illumina-like allele coding and map information (iConvert) [28] and a multi-purpose software for SNP data management and analysis (Plato 2.0) [29].

The database interface is the true core of SNPchiMp v.3, and of course was present in the other two versions of the tool. Although background functionalities were completely rebuilt, special attention was taken to maintain user experience as similar as possible to earlier versions. User access to the database is driven by two functions, specifically designed to efficiently query either all SNP array information (“Download” menu) or a subset of SNPs (“Browse” menu). After selecting the desired species, a series of dialog forms and JQuery-Javascript functions (i.e. for radio and box buttons) help users perform the required query of the database in a user-friendly fashion. No SQL coding skills or knowledge of the underlying database is required. User choices consist of the selection of the desired SNP array, genome assembly and the level of detail required for the expected results. For both download and browse functions a subset of the first 25 rows of the query results is shown in a preview table. After being previewed, the resulting dataset can be downloaded by simply clicking on a “download” button.

Three specific features are available when the Browse feature is used to query a subset of SNPs. Firstly, the preview page with results that contains links to related external resources/databases. On each row of the SNP table, two links for SNP specific Ensembl Locations/Variations and NCBI dbSNP pages are displayed. The second feature included in this section, which was not available in earlier version of this tool, was included in response to requests from the animal genetics community, and is the possibility to perform a straightforward SNP array specific annotation. SNPchiMp v.3 facilitates this by direct use of the Ensembl BioMart resources, using a “URL based access” interface to the Biomart Central Portal [30]. The SNPchiMp v.3 interface prepares the Biomart URL, containing the specified query parameters, in order to visualize the required subset of data. This avoids any need for programming or specific knowledge of the data-mining tools involved. More advanced users can refine their queries with an additional set of options as required. The third feature facilitates the direct interrogation of Ensembl’s Variant Effect Predictor (VeP) tool using a REpresentational State Transfer (REST) interface. A Python CGI script was created to submit the data to Ensembl’s VeP REST API and visualize the results within the SNPchiMp v.3 interface. Results are displayed in a table where Ensembl VeP annotations are reported for each SNP. This solution ensures the stability of the service provided, as it avoids the need to install and update data and/or software for each new Ensembl data release as the REST interface automatically accesses the most up-to-date Ensembl version.

## Conclusions

The need for SNP data standardization and integration is underappreciated. Here we present a tool that allows users to avoid performing the long and time-consuming series of steps required to integrate, standardize, and annotate SNP array data in the major livestock species.

From its initial release [23], this tool was completely rebuilt both on the back- and on the front-end, in order to enhance manageability and user-friendliness. SNPchiMp v.3 now considers six livestock species, for a total of 23 SNP arrays and allows direct and easy annotation of SNP array data in multiple species using Ensembl resources. It also provides standalone software for basic SNP data management. Its maintenance and constant update is ensured by the strong collaboration between SNP array producers, International Genome Consortia and the animal genetics community. In recognition of the open-access use of Ensembl resources, SNPchiMp v.3 was officially credited as an Ensembl E!mpowered tool [31].

## Availability and requirements

**Project name:** SNPchiMp v.3

**Project home page:**http://bioinformatics.tecnoparco.org/SNPchimp

**Operating system(s):** Platform independent

**Programming language:** PHP, JQuery-Javascript, SQL, Python

**Other requirements:** none

**License:** GNU GPL

**Any restrictions to use by non-academics:** none
